# A hybrid CNN-RNN approach for survival analysis in a Lung Cancer Screening study

**DOI:** 10.1016/j.heliyon.2023.e18695

**Published:** 2023-08-03

**Authors:** Yaozhi Lu, Shahab Aslani, An Zhao, Ahmed Shahin, David Barber, Mark Emberton, Daniel C. Alexander, Joseph Jacob

**Affiliations:** a Centre for Medical Image Computing, University College London, UK; b Department of Computer Science, University College London, UK; c Department of Respiratory Medicine, University College London, UK; d Centre for Artificial Intelligence, University College London, UK; e Division of Surgery and Interventional Science, University College London, UK

**Keywords:** Computed tomography, Lung, Deep learning, Computer vision, Saliency map, Longitudinal data, Cox regression

## Abstract

In this study, we present a hybrid CNN-RNN approach to investigate long-term survival of subjects in a lung cancer screening study. Subjects who died of cardiovascular and respiratory causes were identified whereby the CNN model was used to capture imaging features in the CT scans and the RNN model was used to investigate time series and thus global information. To account for heterogeneity in patients' follow-up times, two different variants of LSTM models were evaluated, each incorporating different strategies to address irregularities in follow-up time. The models were trained on subjects who underwent cardiovascular and respiratory deaths and a control cohort matched to participant age, gender, and smoking history. The combined model can achieve an AUC of 0.76 which outperforms humans at cardiovascular mortality prediction. The corresponding F1 and Matthews Correlation Coefficient are 0.63 and 0.42 respectively. The generalisability of the model is further validated on an ‘external’ cohort. The same models were applied to survival analysis with the Cox Proportional Hazard model. It was demonstrated that incorporating the follow-up history can lead to improvement in survival prediction. The Cox neural network can achieve an IPCW C-index of 0.75 on the internal dataset and 0.69 on an external dataset. Delineating subjects at increased risk of cardiorespiratory mortality can alert clinicians to request further more detailed functional or imaging studies to improve the assessment of cardiorespiratory disease burden. Such strategies may uncover unsuspected and under-recognised pathologies thereby potentially reducing patient morbidity.

## Highlights

• A CRNN approach to predict long-term survival using the lung cancer screening patients' imaging history.
• Predict a patient's cardiorespiratory mortality risk, thus suggesting timely personalised interventions.
• Improving Lung Cancer Screening study's cost-effectiveness and health benefits.

## 1 Introduction

### 1.1 Overview

Cardiac and respiratory illnesses are the leading causes of mortality globally [1], [2], especially amongst older age groups. Compounded with the ageing global population [3], this translates to an increased number of patients with multimorbid conditions and causes spiralling pressure on healthcare services. Responding to such growing healthcare needs requires cost-effective approaches to early disease detection. Early detection allows timely intervention before diseases become irreversible.

Various large-scale Lung Cancer Screening (LCS) studies, such as NELSON (Dutch-Belgian) [4], SUMMIT (UK) [5], the National Lung Screening Trial (NLST, US) [6], [7], have been set up to improve the detection of early lung cancer in high-risk populations. Screening involves annual computed tomography (CT) imaging and various clinical measurements (e.g. lung spirometry). LCS with CT imaging has been demonstrated to reduce lung cancer mortality in the NLST effectively [6], [7], [8] and NELSON studies [4].

A critical and currently underutilised benefit of imaging acquired as part of LCS is the potential to detect underdiagnosed illnesses in LCS populations. For example, Cardiovascular Disease (CVD) shares the same risk factors as lung cancer. Accordingly, cardiovascular risk detection could be improved by a detailed interrogation of LCS imaging. Similarly, detecting early respiratory pathologies that influence survival could also be improved. Currently, lung cancer detection is typically the radiologist's overarching aim, but improving the detection of cardio-respiratory morbidity could enhance LCS's cost-effectiveness and overall health benefits.

Rather than analysing images in isolation, this study proposes a hybrid CNN-RNN approach to fully utilise both the imaging and temporal information to predict long-term survival outcomes in a LCS cohort. In turn, this may allow early interventions that may prevent or delay the occurrence of adverse cardio-respiratory health events, thereby prolonging a patient's life expectancy.

### 1.2 Literature review

Lung cancer and Cardiovascular Diseases (CVD) share several similar risk factors, including smoking (both active and passive) and exposure to fine particulates from air pollution [9]. Though the pathophysiological mechanisms differ, it has been shown that smoking leads to increased mortality risk from lung cancer, lung fibrosis, chronic obstructive pulmonary disease and CVD [9], [10]. It is, therefore, logical that patient cohorts enriched with heavy smokers, such as LCS studies [4], [5], [6] can be used to develop prediction models of CVD-related mortality.

Recently, attempts have been made to predict CVD-related mortality in the NLST cohort [11], [12], [13]. As demonstrated in van Velzen et al. 2019 [11], a Convolutional Autoencoder (CAE) was trained to derive abstract image features. Then, the extracted features were fed into three separate classifiers to predict CVD-related mortality. The CAE encoded the automatically extracted 3D LDCT volume around the heart and exported the image features to the subsequent classifiers. The support vector machine classifier achieved performance in the Area under ROC curve (AUC) of 0.72. Though the study recognised the value of using clinical information for prediction, including handcrafted variables such as the Coronary Artery Calcium (CAC) score [13], [14], [15], which is a known predictor of CVD, such information was not utilised in making predictions. Instead, the study demonstrated that it is possible to predict CVD-related mortality from LDCT scans alone.

Predicting CVD-related mortality was further improved by Guo et al. 2020 [12]. A multimodal approach was adopted in this study, where models incorporated both LDCT imaging information and handcrafted features to make mortality predictions. When the contributions of the imaging features and clinical data were optimised, this approach improved the AUC performance to 0.82. Both methods show improvement over human performance in this regard. In fact, as reported by Guo et al., visual inspection of the coronary artery calcium measured by a radiologist could only achieve performance with an AUC of 0.64.

Instead of examining medical images in isolation without their global context, a few recent studies considered adopting a hybrid model architecture to analyse the imaging and temporal features in a patient's data. Thus, hybrid models, which have a CNN portion to investigate the medical imaging features while a RNN portion to keep track of the temporal information, had been adopted to examine retinal video [16], spectrogram [17], chest radiograph [18], and computed tomography [19] etc. Thus, global temporal information embedded in the follow-up scans can be fully utilised. In the above-mentioned cases, the hybrid models outperformed their CNN-only counterparts, which naturally limited the contextual information from the patient's follow-up history. Thus, the findings illustrate the superior performance of using time-series data over a single snapshot in making the diagnosis.

Given the unavoidable heterogeneous follow-up times in clinical practices, various studies [18], [19], [20] had attempted to incorporate the interval variation in their analysis. Instead of adopting the implicit homogeneous interval assumption in the LSTM model, various approaches [18], [19], [20] of adding time-weighted terms to accentuate the temporal irregularities are proposed. For example, Santeramo et al. 2018 [18] proposed additional interval time-related weights to the LSTM gates calculation, while Gao et al. 2019 [19] proposed using the Temporal Emphasis Modules (TEM) to emphasise the more recent scans.

An alternative and more informative approach to evaluating survival would be the Cox Proportional Hazard (PH) model [21] which examines the effect of covariates on the patient's time to death. Katzman et al. 2018 [22] proposed, DeepSurv, a Cox Proportional Hazard neural network to examine clinical data to help provide a personalised approach to predict a patient's survival time. A few studies [23], [24] have applied a similar approach to survival analysis using medical images. In particular, Shahin et al. 2022 [24] performed survival analysis in an Idiopathic Pulmonary Fibrosis cohort with a modified Residual Network and an equivalent Cox PH loss function.

In our previous work, Lu et al. 2021 [25], 3D-CNN-based models were deployed to inspect the latest screening CT scan and correlate the imaging features to long-term survival status. A multimodal approach utilising the clinical information was also attempted. However, as discussed in the literature review, such approaches under-utilise the global information in the LCS study and discard an individual's speed of disease progression. Therefore, in this study, we adopted a hybrid CNN-RNN approach to treat the patients' follow-up scans as time series data and combined the local and global information for improving risk prediction for cardiorespiratory mortality. As can be demonstrated, the hybrid approach can enhance model performance and, in particular, improve their generalisability. When applied to the prediction of time to cardiorespiratory death, the application of such a method may help direct timely early medical tests or therapeutic interventions that could prevent or lessen adverse cardio-respiratory events, thus reducing morbidity and mortality.

The key contributions of the study are as follows:

• A hybrid 4D CRNN approach to predict long-term cardiorespiratory mortality using the patients' longitudinal LCS imaging history.
• Prediction of increased cardiorespiratory mortality could suggest those subjects in whom timely personalised interventions may help reduce morbidity and mortality.

## 2 Methods

### 2.1 Dataset selection and split by screening centres

The National Lung Screening Trial was a lung cancer screening study conducted with 33 US screening centres from 2002 to 2007 [6], [7], [8]. 53,454 heavy smokers aged 55-74 at high risk for developing lung cancer were recruited. The exclusion criteria for the study [6] include the presence of other forms of cancer, unexplained weight loss, and acute respiratory infections such as pneumonia etc. For our study, we were provided with a subset of 15,000 patients' imaging data comprising three annual screening CT attendances, denoted as T0-T2. The participants' survival status and the cause of death were ascertained through the evaluation of death certificate ICD-10 (International Classification of Diseases, 10th edition) codes. In the LDCT branch, the leading cause of death (as of the end of 2009) was cardiovascular disease (26.1%), followed by lung cancer (22.9%) and then other types of cancer (22.3%) [7]. One of the studies targeting the 2009 endpoint was conducted by Chao et al. 2021 [13], which aims to analyse cardiovascular disease survival.

In our study, the participant's survival status in 2015 was chosen as the ground-truth label, and the follow-up times were adjusted with respect to the latest scan (i.e. T2). The targeted causes of death were cardiac^1^ and respiratory diseases,^2^ while excluding other mortality causes (i.e. lung cancer). We primarily focused on predicting cardiorespiratory mortality as in lung cancer screening populations a high smoking burden, increased age and socioeconomic factors combine to increase the risk of damage to the respiratory and cardiovascular systems. Damage to either the cardiac or respiratory system in turn accentuates damage in the other system. Cause of death labels used in clinical studies are often the primary cause of mortality i.e. myocardial infarction. Yet a subject in a lung cancer screening study will have numerous secondary causes of mortality such as emphysema, airway disease and/or lung fibrosis. We aimed to develop a real-world model that can help predict subjects with an increased risk of cardiorespiratory mortality, and capturing these features of respiratory damage is crucial to this endeavour. Similarly, a subject dying with a cause of death given as lung fibrosis will have cardiac disease which would be a secondary cause of mortality. Therefore it was our belief that trying to distinguish cardiac and respiratory mortality created an artificial separation in the common pathophysiological mechanisms caused by smoking and ageing in both organ systems.

Only cases with all three screening CTs (i.e. T0-2) were included to avoid bias introduced by patients who left the trial early for unknown reasons. Only CT scan thickness in the axial plane of less than 2.5 mm was analysed. A radiologist reviewed the corresponding CT images to exclude cases with imaging artefacts and anatomical biases (i.e. severe forms of thoracic spinal scoliosis). The patients with cardiac and respiratory deaths were age, gender, and smoking history matched in a 1:2 ratio with a control population of survivors.

2154 patients from 32 NLST screening centres meet the eligibility criteria mentioned above. CT scans from 26 of the 32 centres were used as the ‘internal’ training-validating-testing dataset (n=1,869). Scans from the remaining 6 centres were kept aside as the ‘external’ dataset (n=285) for later evaluation of the generalisability of the models on heterogeneous imaging protocols and equipment (i.e. out-of-distribution data). The composition of the datasets is tabulated in Table 1. The current combination of the centres in the subsets allows the maximum number of scans to be used.

**Table 1:** Composition of the datasets.

Dataset		Survivor	Non-survivor		Total
			Cardiac	Respiratory	
Internal	CV fold 1	208	47	57	312
	CV fold 2	208	56	48	312
	CV fold 3	208	59	45	312
	CV fold 4	208	62	42	312
	CV fold 5	208	50	54	312
	Test	206	53	50	309

External	Test	190	47	48	285

Total		1436	374	344	2154

**Figure 1:**
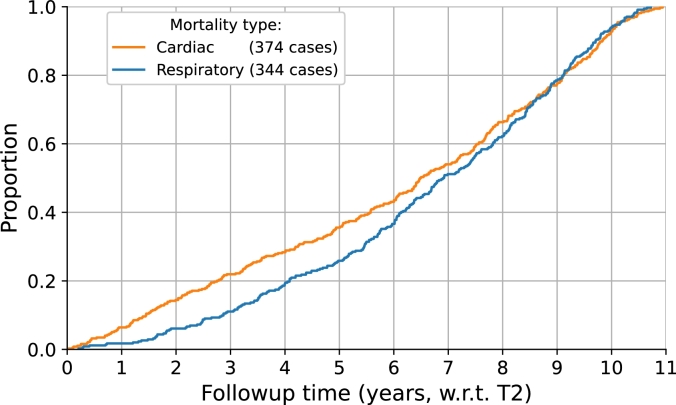
Distribution of the non-survivors' follow-up time.

As shown in Table 1, after a random split, 1560 cases from the internal dataset (n=1,869) were used for a 5-fold cross-validation while the remaining 309 cases were used as an internal test set to assess model performance during training. The composition of the subsets was kept the same throughout the study. The trained models were evaluated on 285 patients' scans in the external test. Additionally, the relative proportions of the two causes of mortality, which are also presented in Table 1, were maintained across the internal and external datasets. The time to death of the non-survivors from the date of the last screening point is illustrated in Fig. 1.

### 2.2 Lung CT-volume preprocessing

**Figure 2:**
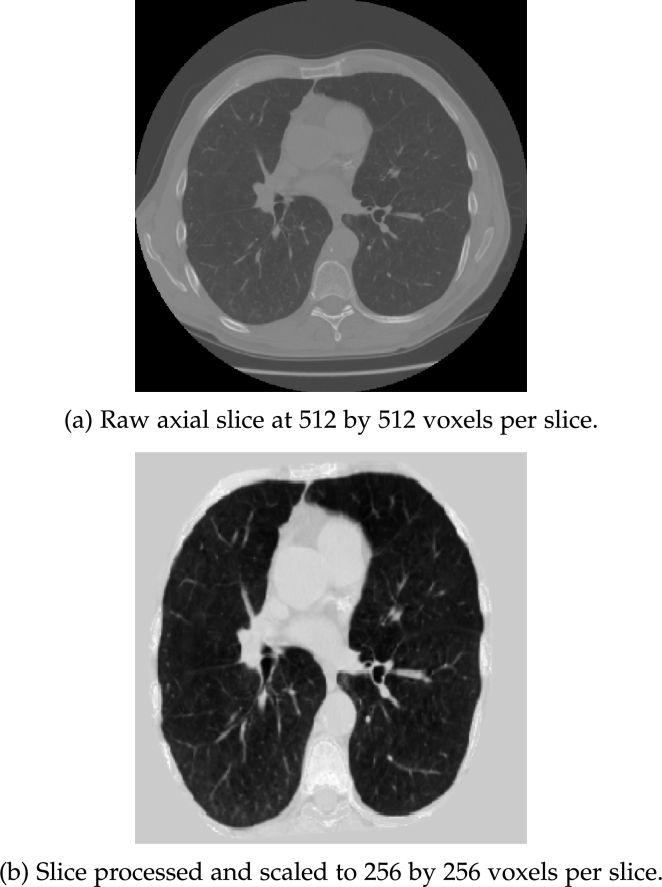
CT volume pre-processing.

Similar to the previous study [25], the primary imaging processing pipeline consisted of a modified pre-processing approach from Liao et al. [26]. The CT image was filtered with a Gaussian filter for each axial slice, and a -600 Hounsfield Unit (HU) threshold was applied to create the binarised slice. The absolute size and eccentricity of connected components were then used to filter out small components and imaging noise. The resulting 3D volumes were then filtered by their size (0.68 - 7.5 L) and distance to the centre of the scan. The results were joined to create the approximate lung mask. Morphological transformations, i.e. erosion, dilation, and convex-hull calculation, were performed to further separate the result into the left and right lung masks. An additional convex-hull analysis was performed on the joint lung masks to include the cardiac region in the final results. The Hounsfield Unit (HU) range was clipped to an interval between -1200 HU to 600 HU. The range was linearly normalised to between 0 and 255. The regions outside the masks corresponding to the surrounding tissue were filled with an average value of 170. The pre-processing pipeline was applied over all axial layers to extract the thorax region in 3D. The processed 3D CT volumes were interpolated into the shape of 256 by 256 by 128. An example case is illustrated in Fig. 2 where Fig. 2(a) is a CT scan in DICOM format while Fig. 2(b) is the pre-processed input to the neural networks.

**Figure 3:**
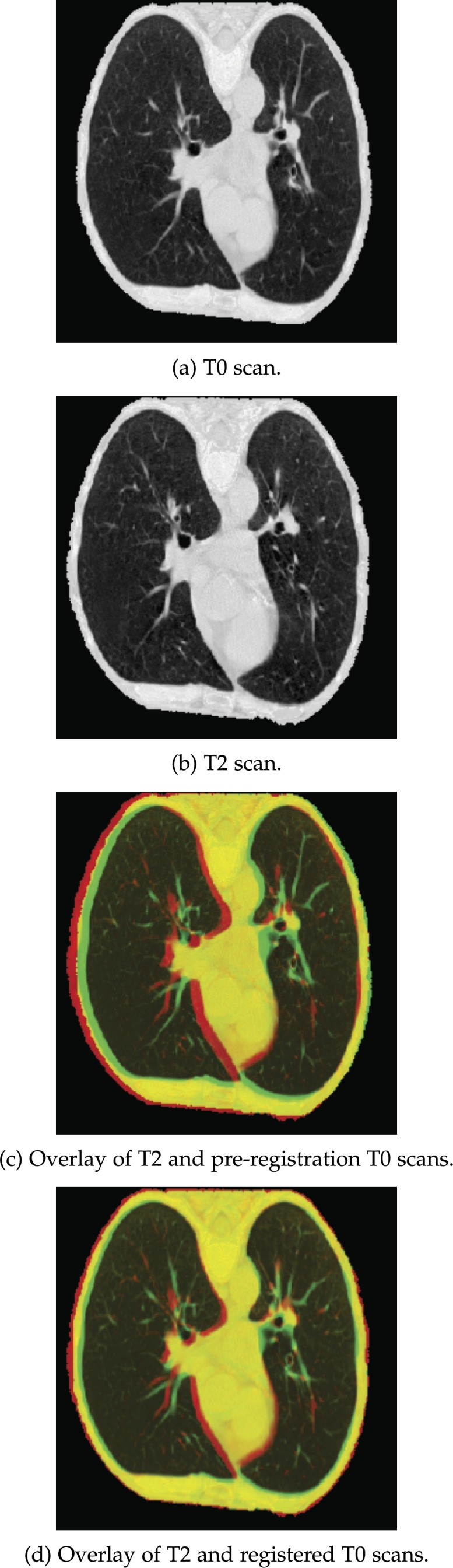
3D affine registration demonstrated on 2D slices.

The aforementioned imaging pre-processing pipeline does not explicitly perform image registration. However, it does align the image by utilising the joint mask. To assess whether image registration can lead to performance improvement, particularly for the time-series models, 3D affine registration was attempted for one of the sub-studies. In particular, the scans from the two earlier screening points, T0 and T1, were registered to the most recent T2 scan through the DIPY^3^ (v1.5.0) library [27]. The example results of the 3D affine registration are illustrated in Fig. 3. The T0 scan is illustrated in Fig. 3(a) while the T2 scan of the same patient is shown in Fig. 3(b). Comparing Fig. 3(c) to Fig. 3(d), it can be seen that the additional processing has minimised the difference in position and orientation between the T0 scan to the target T2 scan.

### 2.3 Models

#### 2.3.1 CNN models

The deep-learning-based models in this study were based on a 3D implementation of the ResNet [28][29]. A 10-layer implementation of the 3D ResNet was used as the backbone for both the CNN models and the subsequent CRNN models in this study. The choice of the CNN network depth reflects the trade-off between performance and model size. The pre-trained weights from Chen et al. [29], initially optimised for medical imaging (CT images) segmentation tasks, were used as initial weights during training. The output from the 3D ResNet backbone was converted to a 1D tensor after passing through a global adaptive average pooling layer. It was then passed through two subsequent fully-connected layers (including dropout with p=0.5 and ReLU activation) with 512 and 32 neurons, respectively.

The CNN models were trained with the latest scan, i.e. NLST T2 scan. The 3D CT volumes were interpolated into 256 by 256 by 128 tensors and fed into the networks. The 2015 survival outcome was used as the ground truth label for the classification task between survivors and non-survivors.

#### 2.3.2 CRNN models

**Figure 4:**
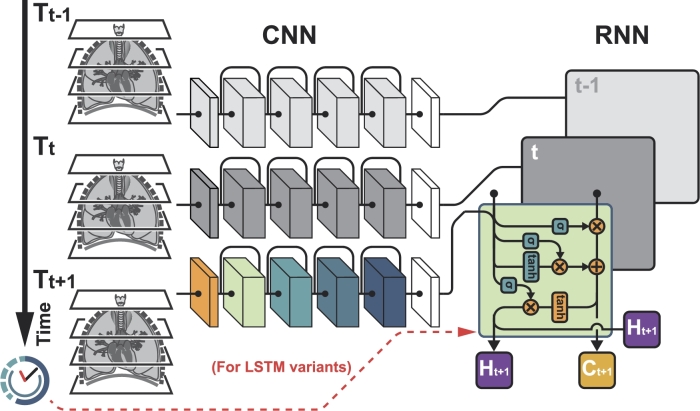
Schematic diagram of the CRNN model.

The two sides of the CRNN model, as illustrated in Fig. 4, were trained independently from each other. The model parameters from the trained CNN model were inherited by the CNN side of the model and were not optimised during the CRNN training. In contrast, the RNN (i.e. LSTM) side, which feeds in the spatial /imaging feature from the CNN side, was optimised during CRNN training. In other words, the CNN backbone was used to capture the local imaging features from each CT scan, while the LSTM model was utilised to capture the global time-series information embedded in the patient's entire follow-up history. The output from the LSTM model was passed through one fully-connected layer (including dropout with p=0.3 and ReLU activation). The number of neurons in the fully-connect layer matched the LSTM's hidden state (hx) size, a tunable hyperparameter.

For each patient, the interpolated 3D CT volumes were stacked into a 4D tensor, with the temporal dimension (i.e. T0-2) as the additional dimension., All 3 NLST screenings were analysed by the hybrid CRNN model, which can process a variable number of scans in a patient's follow-up history. Thus, in this study, the input to the CRNN network was in the form of 256 by 256 by 128 by 3 tensors.

#### 2.3.3 LSTM model comparison

It is important to note that the vanilla LSTM implementation implicitly assumes the inputs are equally spaced. Given the inevitable irregular time intervals between screening timepoints that occur in medical studies, two additional LSTM variants, Time-Aware LSTM (TALSTM) [20] and time-modulated LSTM (tLSTM) [18], which explicitly take irregular time intervals between inputs into account were tested.

To account for the influence of non-uniform time intervals, the former model [20] attempts to separate the memory component in LSTM into its long and short-term parts, with the latter's effect disregarded. In contrast, the time-modulated LSTM variant [18] explicitly introduces temporal weights into the LSTM gates update. This is illustrated by the modified LSTM gates formulas. In Eqs. (1) to (4), $f_{t}$, $i_{t}$, $g_{t}$ and $o_{t}$ are the LSTM forget, input, cell, and output gates respectively. $x_{t}$ is the imaging feature at time *t* while $h_{t-1}$ is the hidden state at time t−1 or the initial hidden state. The additional weighted terms, represented as $W\delta_{t}$, introduce the effect of variations in follow-up intervals $\delta_{t}$.

(1) $$f_{t}=\sigma (W_{if}x_{t}+W_{hf}h_{t-1}+W_{tf}\delta_{t}+b_{f})$$

(2) $$i_{t}=\sigma (W_{ii}x_{t}+W_{hi}h_{t-1}+W_{ti}\delta_{t}+b_{i})$$

(3) $$g_{t}=tanh(W_{ig}x_{t}+W_{hg}h_{t-1}+W_{tg}\delta_{t}+b_{g})$$

(4) $$o_{t}=\sigma (W_{io}x_{t}+W_{ho}h_{t-1}+W_{to}\delta_{t}+b_{o})$$

For both variants, as illustrated by the dashed lines in Fig. 4, the temporal information was passed directly into the RNN portion of the model. Pre-processing of the temporal data was implemented in the same manner recommended by the respective studies [18], [20].

#### 2.3.4 Cox Proportional Hazard Network

In addition to performing classification on the long-term survival outcome, a survival outcome prediction based on the Cox Proportional Hazard model [21] was attempted using the same CNN and CRNN networks. In this study, the survival time is right-censored and adjusted with respect to the latest screening point (T2).

In survival analysis, a hazard function, which is shown in Eq. (5), measures the instantaneous risk of event /death (E=1) at time *t* for an individual that has survived beyond time *t*:

(5) $$h(t)=\lim_{△t\to 0}\frac{p(t\leq T<t+△t|T\geq t)}{△t}$$

The Cox PH model attempts to model the hazard function given a patient's baseline data *x*:

(6) $$h(t|x)=h_{0}(t)\cdot e^{h(x)}$$

In Eq. (6), $h_{0}(t)$ is the baseline hazard function and represents the hazard when all the covariates are zero. h(x) in this study is the output of the neural network. To optimise such a network, the negative partial log-likelihood function [22], [24] is implemented:

(7) $$L=-\frac{1}{N_{E=1}}\sum_{i:E_{i}=1}\left(h(x_{i})-\log\sum_{j\in R(T_{i})}e^{h(x_{j})}\right)$$

where $N_{E=1}$ is the total number of patients with an event and $R(T_{i})$ is the set of patients that have not died before patient *i* at time $T_{i}$. The loss function *L* is optimised using stochastic gradient descent.

#### 2.3.5 Experiments

A grid search approach was adopted to tune the hyperparameters in the neural network models. The hyperparameter value that produced the best AUC value was chosen.

Similar to the previous work [25], to counter the class imbalance in the 1:2 matched dataset, a weighted random sampler assigns a sampling probability inversely proportional to the class size was utilised. This approach attempts to create balanced batches during training. Cross entropy loss was adopted as the loss function for the classification model while the Cox Loss in Eq. (7) was adopted for the regression models. The models were trained with Sharpness Aware Minimisation (SAM) [30] with Stochastic Gradient Descent (SGD) as the base optimiser. SAM simultaneously minimises the value and sharpness in the training loss landscape. Thus, it aims to optimise for model parameters within the neighbourhood of low loss and hence improves the generalisability of the model. Adaptive implementation of this optimisation algorithm was also tested, yet it did not improve performance.

As a substudy, an additional set of networks was trained to further differentiate the non-survivor classes into the respective mortality groups, i.e. cardiac-related and respiratory-related. This additional insight provides the clinician with further information as to which areas on the CT need specific consideration. A two-step process is proposed to utilise this model in a clinical setting. Firstly, the main model examines a patient's scans to produce a mortality probability. Secondly, the scans are examined by the additional cause-specific model should the previous output exceed a predefined threshold which is 0.5 in this study. The cause-specific model will then attempt to categorise the site of concern further, and the clinician can perform further checks accordingly. Thus, additional models were trained with only the two non-survivor classes shown in Table 1 and the composition of each subset was kept the same as the main model. The primary CRNN model's training approach was adopted for this substudy.

The classification models were optimised using an initial learning rate of *1E-3*, a cyclic learning rate schedule, momentum of *0.9*, and weight decay (L2 regularisation) of *5E-4*. The regression models differ regarding the initial learning rate, which was set as *5E-3*. Each neural network was trained with a batch size of 24 on a single Nvidia A100 GPU (40GB HBM2) on the UCL CS CMIC cluster. The CNN models were trained with approximately 800 epochs, while the CRNN models were trained with approximately 150 epochs. The deep learning models^4^ were developed in Python (v3.9.5) and PyTorch (v1.9.1).

## 3 Results

### 3.1 Classifying cardiorespiratory mortality

To compare the performance with related studies [11], [12], the models were mainly assessed with the Area Under the Curve (AUC) metric. The average (and standard deviation) of the AUC metrics from the 5-fold cross-validation were used to assess the internal performance of the model. As tabulated in Table 2, the same was done for the F1 score and Matthews Correlation Coefficient (MCC) [31]. To evaluate the model's generalisation ability, the average (and standard deviation) inference performance of the five models on the external dataset was also recorded through the three metrics in Table 2.

**Table 2:** Performance metrics on long term survival prediction.

Study	Model Component(s)		LSTM	5-Fold CV Performance			External Performance		
	CNN	RNN	hx	AUC	F1	MCC	AUC	F1	MCC
A. CNN	ResNet	-	-	0.759 (0.019)	0.626 (0.026)	0.407 (0.047)	0.714 (0.011)	0.578 (0.010)	0.315 (0.021)
B. LSTM	ResNet	LSTM	32	0.763 (0.020)	0.629 (0.031)	0.421 (0.047)	0.731 (0.015)	0.571 (0.021)	0.319 (0.037)
C. LSTM (3d reg)	ResNet	LSTM	32	0.760 (0.017)	0.605 (0.048)	0.404 (0.042)	0.729 (0.014)	0.565 (0.030)	0.324 (0.034)
D. TALSTM	ResNet	TALSTM	32	0.763 (0.019)	0.628 (0.034)	0.423 (0.048)	0.730 (0.015)	0.573 (0.026)	0.326 (0.035)
E. tLSTM	ResNet	tLSTM	64	0.763 (0.018)	0.630 (0.028)	0.419 (0.046)	0.731 (0.014)	0.575 (0.018)	0.320 (0.039)

In the baseline Study A, where the CT scans are analysed in isolation from the rest of the sequence of CT time points, the model reached an average AUC of 0.759, an average F1 of 0.626, and an average MCC of 0.407 on the internal dataset. As expected, the performance in all three metrics deteriorated when evaluated on the external dataset. In comparison, when the global and temporal information from the patient's follow-up was considered in Study B, all three performance metrics on the internal dataset improved over those in Study A. In particular, the Matthews Correlation Coefficient improved by 3.4% from 0.407 to 0.421. The extent of the performance improvement introduced by the hybrid approach is on par with related studies [18][19].

**Figure 5:**
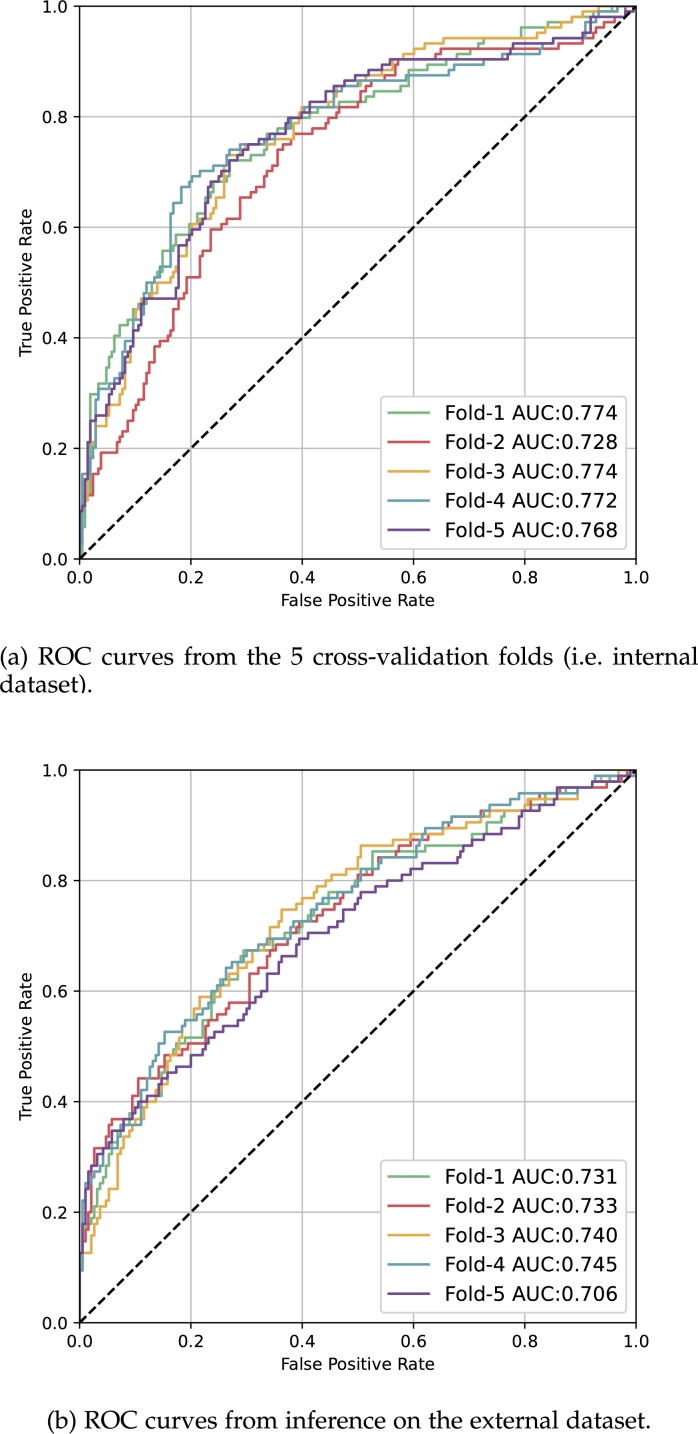
CRNN model (Study B) performance illustrated by Receiver Operating Characteristic (ROC) curves.

More importantly, the generalisation ability of the model, indicated by performance on the external dataset, improved, as illustrated by the increase in AUC performance from 0.714 to 0.731 in Table 2. The corresponding ROC curves for the internal dataset and for the external dataset are shown in Fig. 5(a) and (b) respectively. To assess the statistical significance of the AUC performance between Study A and Study B, DeLong's algorithm [32], [33] was used. In this analysis, the five cross-validation models were treated as an ensemble model with their predicted survival probability averaged. The resulting p-value was found to be less than 0.02.

**Figure 6:**
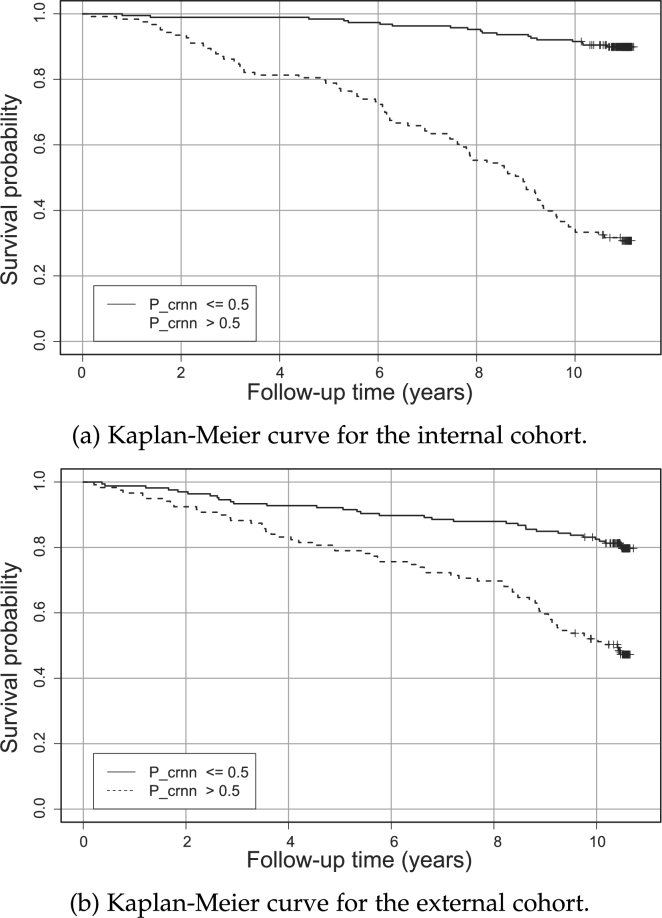
Kaplan Meier survival curve for the predicted non-survival probability (*P*_*crnn*_).

Kaplan-Meier curves were plotted to examine the survival outcomes of the patient cohort using the predicted mortality probability from the ensemble CRNN models in Study B. As illustrated in the Kaplan-Meier curves in Fig. 6(a) and (b), which depict the survival outcomes over time, the patients with a mortality probability $P_{crnn}$ higher than 0.5 have a markedly lower survival rate than their counterparts. This analysis demonstrates the approach's capability to predict long-term survival outcomes.

**Figure 7:**
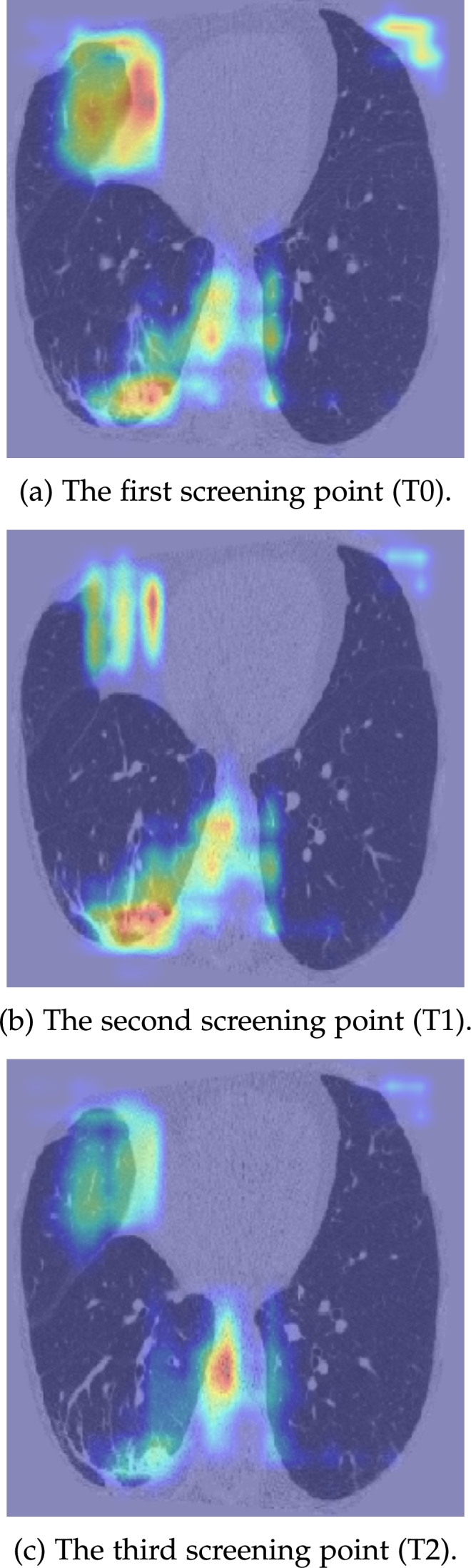
GradCAM overlays of the 3 screening points for a COPD non-survivor.

Study C, where a 3D registered dataset was used to train the combined CRNN model, showed no improvement in performance on internal and external datasets. It also showed that the additional noise introduced by the affine registration did not outweigh the benefits gained. To further investigate the utility of the 3D registration, GradCAMs (Gradient-weighted Class Activation Map) [34] from a Chronic Obstructive Pulmonary Disease (COPD) non-survivor's follow-ups scans are shown. As illustrated by the GradCAM overlays in Fig. 7(a) to (c), the model is relatively consistent with lesion position over time, without the explicit registration, thereby negating the need for the additional 3D registration.

In Study D and E, where the irregularity in follow-up intervals are explicitly addressed, the performance is comparable to Study B, which implicitly assumes constant intervals between scans. This implies the follow-up interval variation, which has an inter-quartile range of 40 days, provides limited information to the long-term survival prediction task.

### 3.2 Classifying cause-specific cardiorespiratory mortality

The CRNN network in Study B was applied to patients who died from cardiac and respiratory deaths, as shown in Table 3, where deaths were classified into three bands: 0-3 years from the last CT date (i.e. T2); 0-7 years from the last CT date; within 11 years of the last CT date. The model had initially been trained with labels corresponding to known patient deaths 10 years after the first CT, but had not been provided with information about the exact cause of death (i.e. whether cardiac or respiratory related). Any patient predicted by the ensemble model from Study B to have a less than 0.5 mortality probability was classified as a survivor. Any cases passing this probability threshold were separated into the two causes of death by the CRNN model in Study F. The final prediction from this two-step decoupled process was then evaluated against their ground truth label of dead/alive within the relevant time band. Accordingly, the ground truth survival status varied according to the follow-up time band.

**Table 3:** Performance by time range and cause of mortality (Study F).

Class	Band a (death <3.0 yrs)			Band b (death <7.0 yrs)			Band c (death <11.0 yrs)		
	Cases	Sensitivity	Specificity	Cases	Sensitivity	Specificity	Cases	Sensitivity	Specificity
Survivor	2034	0.626	0.744	1776	0.688	0.746	1436	0.791	0.719
NS (Cardiac)	82	0.476	0.771	202	0.510	0.806	374	0.513	0.853
NS (Respiratory)	38	0.737	0.765	176	0.665	0.808	344	0.634	0.861

As shown in Table 3, respiratory mortality within 3 years of the CT scan was predicted with good sensitivity (0.737) and specificity (0.765), with cardiac mortality prediction also showing good specificity (0.771) but poor sensitivity (0.476). Respiratory death prediction showed improved specificity and acceptable sensitivity at time intervals increasingly distant from the last available imaging that could be analysed. Cardiac death prediction however showed poor sensitivity and good specificity across all follow-up time bands. An alternative approach for cause-specific mortality prediction, similar to the one conducted by Chao et al. 2021 [13], would be using separate models and add prior clinical knowledge (e.g. Coronary Artery Calcification score) in the models to predict the specific type of mortality.

### 3.3 Predicting patients' risk through the Cox model

Our final analysis considered discriminating the NLST cohort based on their cardio-respiratory mortality risk. The performance of the Cox-regression-based survival analysis was measured across internal and external cohorts and evaluated using the Inverse Probability of Censored Weights (IPCW) Concordance Index [35] which is modified from the original Concordance Index [36]. The tabulated metrics in Table 4 illustrate that adopting the CRNN model which examines all available follow-up imaging information outperforms the CNN approach which investigates the latest CT scan in isolation. In particular, the IPCW C-index is improved by 4.3% from 0.719 to 0.750 between the two models. Though less pronounced, an improvement of 2.5% in IPCW C-index from 0.673 to 0.690 can be observed in the external dataset. This reaffirms our hypothesis that the hybrid CNN-RNN approach improves model performance by capturing additional global time-series CT features that relate to cardio-respiratory disease progression.

**Table 4:** Regression model performance in terms of average IPCW C-index (and standard deviation).

CoxPH	Model	LSTM	IPCW C-Index	
Study		hx	5-fold CV	External
G. CNN	ResNet	-	0.719 (0.040)	0.673 (0.009)
H. CRNN	ResNet+LSTM	32	0.750 (0.050)	0.690 (0.019)

## 4 Discussion

Our study examined the ability of time-series imaging data to predict cardiorespiratory mortality in subjects participating in a lung cancer screening study. We demonstrate that time series analysis improves mortality prediction when compared to single timepoint data. We demonstrate the high sensitivity and specificity of our model in estimating the likelihood of respiratory deaths at 3 years, and the high specificity for the detection of cardiac-related deaths across follow-up timepoints. We also demonstrate the improved ability of our time-series model to predict the time to cardiorespiratory death using a modified Cox model when compared to single timepoint data.

### 4.1 Survival prediction using both spatial and temporal data

By comparing the performance between Study A and B, in Table 2, it is evident that the inclusion of longitudinal information improves classification performance. When compared to examining CT images in isolation, (Study A where a CNN model is adopted to view the latest CT scan), considering the entire follow-up imaging history of a patient provides a greater global context of the patient's health and/or disease progression. Specifically, the CRNN model improves the internal MCC by 3.4% from 0.407 to 0.421 and the external AUC by 2.4% from 0.714 to 0.731. The extent of the improvement, through the inclusion of temporal information, is consistent with that observed in related studies [18], [19].

Despite the marginal improvement in external F1 and MCC, the irregular time interval model examined in Study D and E showed similar performance to the model evaluated in Study B. This is in contrast to the performance improvement noted in the related studies [18], [19], [20]. It can be argued that this study's classification task, which focuses on long-term survival, is more tolerant to the time interval variation than counterparts, such as the study by Santeramo et al. 2018 [18]. Yet the most likely cause for the comparable performance between models that did and did not consider time intervals is the relatively constant time interval (inter-quartile range of 40 days between CTs) in the NLST study which was a clinical trial. Models trained to examine irregular time intervals on longitudinal data are far more likely to show utility in real-world data where imaging intervals are more varied than would be seen in protocolised clinical trials.

### 4.2 Cause-specific survival outcomes

Our results demonstrate how differently our model treated respiratory versus cardiac deaths in the screening population. The high sensitivity and specificity for 3-year respiratory mortality suggest the model has potential utility in identifying screened patients who might benefit from targeted respiratory interventions over a meaningful time frame for clinical trials.

The low sensitivity of the model for cardiac death prediction may relate to the inherently unpredictable nature of many acute cardiac events. A central challenge of cardiology lies in risk prediction and prevention for populations at risk of adverse cardiovascular events. Whilst calcification can be identified in coronary arteries on non-contrast-enhanced CTs, calcified plaques are the main surrogates for the presence of non-calcified plaques which are more likely to rupture. Yet coronary calcification and non-calcified coronary damage may be very challenging to identify comprehensively on low-dose, non-gated, non-contrast CT scans. The high specificity for cardiac death prediction identified in our study suggests some events can be predicted using imaging and the results are reassuring in the context of a screening population, where false positive identification of disease should be avoided. Analysis of the time-series CTs in a screening cohort with a model such as ours could help rule-in subjects who would warrant a more definitive evaluation of cardiac disease with for example a contrast-enhanced, gated coronary CT scan.

The improved performance of the Cox regression model when using time-series data confirms our hypothesis that capturing change in imaging features and therefore disease progression, is important when estimating mortality and has clear advantages over single timepoint data. The standard workflow for a radiologist analysing imaging data relies on the assessment of historic imaging to assess the extent of disease progression. Models utilising the additional information available through repeated imaging therefore should be better at estimating disease progression.

## 5 Conclusion and future works

The results shown in our study lead us to the following conclusions and directions for future studies:

1) The comparison between the baseline CNN model and the main CRNN model indicates that capturing both local and global information in a patient's medical history can lead to better model performance and improved generalisation ability. Thus, unsurprisingly, medical images should not be viewed in isolation. Instead, longitudinal information should be captured to monitor disease progression. The next step would be deploying the current hybrid model on contemporaneous lung cancer screening studies. The SUMMIT study [5] provides such an opportunity where the ability to predict cardio-respiratory adverse events could be examined prospectively.
2) The key objective of this study was to highlight subjects with increased risk of cardiovascular mortality. Once identified, it would be up to the clinician to perform more accurate, organ-specific further tests, both function and imaging-based to tease out the presence of cardiac and respiratory damage. It is unrealistic to suppose that a non-contrast low-dose CT as found in lung cancer screening populations can clearly separate cardiac risk from respiratory risk. Our model was not primarily designed to distinguish cardiac from respiratory mortality, as we believe such a separation is not clinically plausible. Instead, our model signposts those subjects in whom further, more powerful tests are warranted to identify groups in whom intervention in the form of preventative therapies (behaviour modification i.e. exercise or smoking cessation; statin and anti-platelet therapy; pneumococcal vaccination) should be implemented or heavily encouraged.
3) It is clear from Study D and E that the limited variation in follow-up intervals across the NLST screening time points did not unduly influence long term survival prediction. It would be important to deploy models that consider irregular time interval acquisitions on non-protocolised real-world data to gauge the influence of irregular scanning intervals on estimations of disease progression.
4) Simultaneously, it would be valuable to investigate the segmentation of the heart region and airways as an avenue to enhance the model's performance by incorporating supplementary information.
5) An alternative to the proposed CRNN model would be a Vision Transformer (ViT) [37]. A transformer-based architecture was proposed by Sarasua et al. 2021 [38] to model spatio-temporal neuroanatomical changes in a patient's left hippocampus. To account for missing follow-up imaging timepoints, padding was applied to the sequences. We would therefore like to explore a time-dependent variant to our model to account for temporal heterogeneity.

## Declaration of Competing Interest

There is no conflict of interest.

## Data Availability

The use of the NLST dataset is approved by the National Cancer Institute (NCI), under Project ID NLST-681.

## Appendix A Supplementary material

MMC 1 Source code for the CRNN model. MMC 1

MMC 2 Approval from the National Cancer Institute (NCI) for using the NLST dataset in this study. MMC 2

## Footnotes

1 ICD-10 codes: I10-I52.

2 ICD-10 codes: J00-J99.

3 The total processing time for 2154 patients, when fully parallelised on an Intel i7-10700K CPU, is approximately 20 hours while running on a SSD.

4 The code repository is available at: https://github.com/aeroelasticitylu/LRsurvival_CRNN.
