# Supplementary material for: A hybrid CNN-RNN approach for survival analysis in a Lung Cancer Screening study
Source: Heliyon. 2023 Aug 3;9(8):e18695. doi: 10.1016/j.heliyon.2023.e18695 (PMC10432611; doi:10.1016/j.heliyon.2023.e18695)
Supplement: MMC 2 — Approval from the National Cancer Institute (NCI) for using the NLST dataset in this study. [file mmc2.pdf]

NATIONAL CANCER INSTITUTE
Cancer Data Access System

Deep learning mortality prediction in NLST data

Principal Investigator

Name
Joseph Jacob

Degrees
F.R.C.R., M.D.(Res).,

Institution
University College London

Position Title
Wellcome Trust Fellow

Email
j.jacob@ucl.ac.uk

About this CDAS Project

Study
NLST (Learn more about this study)

Project ID
NLST-681

Initial CDAS Request Approval
Jun 26, 2020

Title
Deep learning mortality prediction in NLST data

Summary
In patients undergoing lung cancer screening, only 1-2% have lung cancer. The majority of patients are dying of other causes. These may relate to suspected damage in the heart for example, or unsuspected lesions on imaging. Deep learning methods could help elucidate features on CT imaging that link to mortality in lung cancer screening populations.

Aims
Use deep learning computer algorithms to identify features on NLST imaging that link to long-term mortality.

Collaborators
Prof Daniel Alexander, UCL
Prof Geoff Parker, UCL
Mr Moucheng Xu, UCL

Dr Cheung Wing Keung, UCL
Mr Ashkan Pakzad, UCL
Dr Arjun Nair, UCL
Dr Eyjolfur Gudmundsson, UCL
Dr Shahab Aslani
Dr Yaozhi Lu
Mr John McCabe
Mr Bojidar Rangelov
